# A case of synchronous bilateral lung cancers: EML4-ALK positive adenocarcinoma in the right lung and adenocarcinoma in situ (the former bronchioloalveolar carcinoma) in the left lung

**DOI:** 10.1186/1471-2466-13-25

**Published:** 2013-04-25

**Authors:** Ikuo Matsuda, Kengo Takeuchi, Shinjiro Mizuguchi, Masahide Kaji, Kayo Ueda, Kazuhiro Teramura, Seiichi Hirota

**Affiliations:** 1 Department of Surgical Pathology, Hyogo College of Medicine, Hyogo 663-8501, Japan; 2 Pathology Project for Molecular Targets, the Cancer Institute, Japanese Foundation for Cancer Research, Tokyo 135-8550, Japan; 3 Department of Surgery, Yodogawa Christian Hospital, Osaka 533-0032, Japan; 4 Department of Pathology, Yodogawa Christian Hospital, Osaka 533-0032, Japan

**Keywords:** EML4-ALK, Lung adenocarcinoma, Adenocarcinoma in situ

## Abstract

**Background:**

Recently it has been revealed that lung adenocarcinomas with distinct gene mutations or fusions are associated with particular histopathological entities. For example, epidermal growth factor receptor (EGFR) gene mutations are often associated with well differentiated adenocarcinoma of the lung with bronchioloalveolar pattern. On the other hand, echinoderm microtubule-associated protein-like 4 (EML4)-anaplastic lymphoma kinase (ALK) fusion gene in a subset of lung adenocarcinoma is related to mucinous cribriform histology.

**Case presentation:**

Reported herein is a case of synchronous EML4-ALK positive lung adenocarcinoma and adenocarcinoma in situ in the bilateral lungs of a 55-year-old Japanese woman. The woman had EML4-ALK positive lung adenocarcinoma in the right lower lung while adenocarcinoma in situ in the left upper lung, which was EML4-ALK negative.

**Conclusion:**

To our knowledge, this is the first report of synchronous, bilateral lung adenocarcinomas composed of EML4-ALK positive and negative ones.

## Background

Adenocarcinomas of the lung comprise a group of diseases with heterogeneous clinicopathological characteristics [1,2]. Histopathologically, adenocarcinoma of the lung is composed of a subtype or mixture of subtypes, including adenocarcinoma in situ (the former bronchioloalveolar), papillary, or acinar ones [1,2].

Recent advances in molecular genomic analyses of lung adenocarcinoma specimens have revealed recurrent association of distinct gene mutations or fusions with particular clinicopathological entities [3]. For example, constitutively active mutations of epidermal growth factor receptor (EGFR) gene are often associated with well differentiated adenocarcinoma of the lung showing bronchioloalveolar pattern. Furthermore, using a functional cloning approach with foci-forming assay, Soda et al. [4] discovered echinoderm microtubule-associated protein-like 4 (EML4)-anaplastic lymphoma kinase (ALK) fusion gene in a subset of lung adenocarcinoma. EML4-ALK positive lung adenocarcinoma typically occurs in young subjects with non- or low smoking habits [5,6]. Histologically, mucinous cribriform pattern is shown to be frequently associated with EML4-ALK positive lung adenocarcinoma [5-7].

Following this breakthrough, a number of kinase gene fusions have been identified in subsets of adenocarcinoma of the lung, the examples of which include RET or ROS1 fusions [7-9]. Most of these fusion genes contained genes for tyrosine kinases as a fusion partner, which are associated with constitutive (ligand-independent) activities.

Since the activities of these fused kinases and mutated kinases are shown to be tumorigenic in cell culture systems and/or transgenic mice, these kinases are promising candidates for therapeutic targets. In fact, small molecule tyrosine kinase inhibitors have shown dramatic therapeutic effects on subsets of lung adenocarcinoma, once oncogenic tyrosine kinases of the cancers are identified. Examples include gefitinib for EGFR mutation-positive lung adenocarcinoma and crizotinib for EML4-ALK positive lung adenocarcinoma.

With regard to these advances in molecular phenotyping, identification of particular histopathological subtypes of lung adenocarcinomas, including bronchioloalveolar pattern or mucinous cribriform pattern, will give important clues for therapeutically tractable genomic changes, such as EGFR gene mutations or EML4-ALK fusion gene.

Reported herein is a case of synchronous EML4-ALK positive lung adenocarcinoma and adenocarcinoma in situ in the bilateral lungs of a 55-year-old Japanese woman. The woman had EML4-ALK positive lung adenocarcinoma in the right lower lung while adenocarcinoma in situ in the left upper lung.

## Case presentation

**Figure 1:**
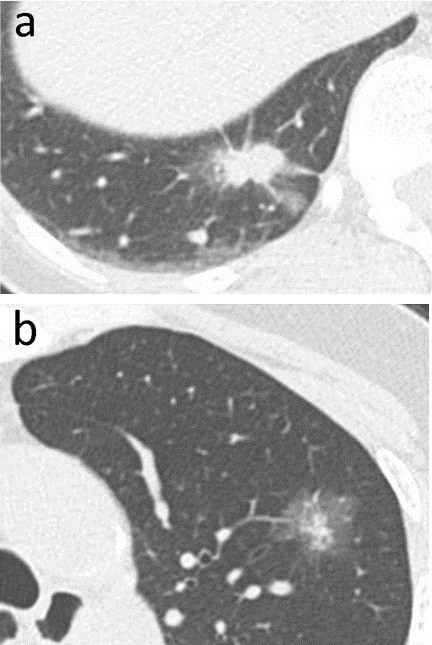
**Preoperative chest CT image.** It reveals a 38×12 mm stellate-shaped mass in the right lower lobe (**a**) and a 15×10 mm ground-glass opacity shadow in the left upper lobe of the lung (**b**).

A 55-year-old Japanese woman was referred to Yodogawa Christian Hospital because of incidental finding of an abnormal shadow in the chest X-ray. She was a never-smoker. Chest computerized tomography (CT) examination revealed a 38×12 mm-sized stellate-shaped mass in the right lower lobe (Figure 1a) and a 15×10 mm ground-glass opacity shadow in the left upper lobe of the lung (Figure 1b). Apparently no enlargement of mediastinal lymph nodes were detected on the CT. Endoscopic biopsy of the left lung tumor showed adenocarcinoma (data not shown). Right lower lobectomy and left upper segmentectomy were performed to resect both mass. Cut surface of the resected tumors revealed a gray stellate-shaped mass in the right lung and a white and yellowish mass with relatively clear border in the left lung (data not shown).

The tissue was fixed in 10% buffered formalin and embedded in paraffin. Three-micrometer-thick sections were stained with hematoxylin and eosin (HE). Immunohistochemistry was performed on representative sections with appropriate antigen retrieval. A primary antibody against ALK was purchased from NICHIREI (NICHIREI 413681; clone #5A4) and the immunostained signal was visualized using I-VIEW DAB universal kit (Roche diagnostics, Switzerland). For mutational analysis, genomic DNA was extracted from formalin-fixed, paraffin-embedded tissue sections using Genomic DNA Extraction Kit (Qiagen, Hilden, Germany). DNA sequencing for EGFR gene mutation was performed as described [10] with minor modifications. Fluorescent in situ hybridization (FISH) analysis using probes for EML4 and ALK genes was performed as described previously [7].

**Figure 2:**
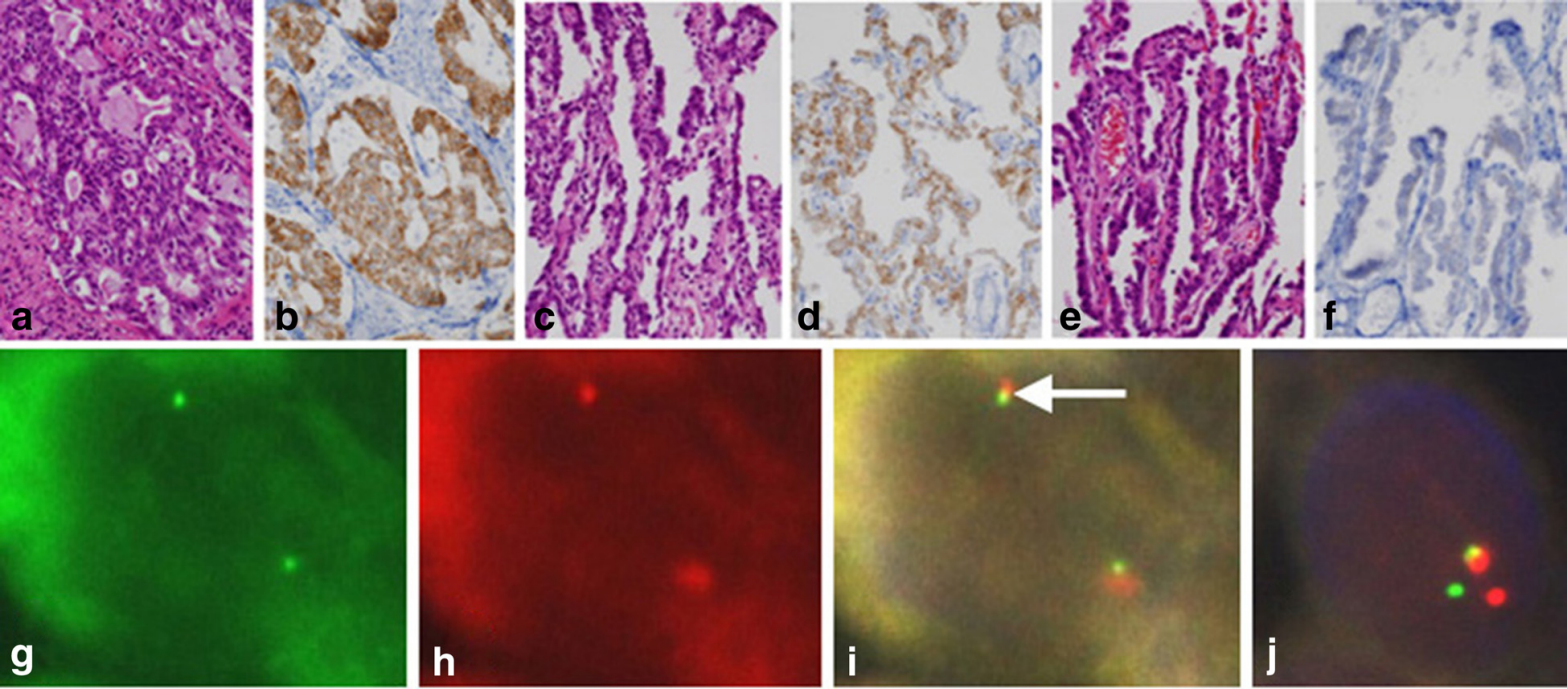
**Histological and FISH analysis.** Microscopic and immunohistochemical examination of the right lung cancer reveals a mucinous cribriform pattern (**a**) (HE stain, ×200), which is positive for ALK (**b**) (immunohistochemical staining, ×200). The right lung cancer also contains a bronchioloalveolar pattern (**c**) (HE stain, ×200), which is again positive for ALK (**d**) (immunohistochemical staining, ×200). On the other hand, the left lung cancer is composed singly of a bronchioloalveolar pattern (**e**) (HE stain, ×200), which is negative for ALK (**f**) (immunohistochemical staining, ×200). FISH analyses revealed EML4-ALK fusion gene in the tumor of the right lung (**g-j**). FISH signals in a single tumor cell are shown. (**g**)-(**i**) EML4-ALK fusion FISH. The signals for ALK gene (**g**) and EML4 gene (**h**) are indicated by green and red dots, respectively. The signal for EML4-ALK fusion gene is indicated by a white arrow (**i**). (**j**) ALK split FISH. The signals for 5′ and 3′ probe for ALK gene are indicated by green and red dots, respectively. Split of the green and the red dots is consistent with the presence of EML4-ALK fusion gene.

Microscopic examination of the resected right lower lobe revealed a heterogenous adenocarcinoma composed of a mucinous cribriform tumor (Figure 2a) and lepidic growth (adenocarcinoma in situ) pattern (Figure 2c). At low-power microscopy, these two components were seen adjacent to each other (data not shown). As described above, the mucinous cribriform histology of the HE stained specimen made us suspicious of EML4-ALK positive lung adenocarcinoma [5-7]. To examine this possibility, immunohistochemical analysis was performed. At low-power microscopy, the tumor was homogeneously stained positive for ALK (data not shown). At high-power microscopy, the tumor cells were positively stained for ALK (Figures 2b,d). Fusion and split FISH analyses using probes for EML4 and ALK genes confirmed that the adenocarcinoma of the right lung was indeed EML4-ALK positive (Figures 2g-j). We confirmed that the EML4-ALK positive adenocarcinoma has no mutations in exons 18, 19, 20, 21 of EGFR gene (data not shown).

On the other hand, the resected left upper lobe specimen contained a adenocarcinoma in situ (Figure 2e). This tumor was negative for ALK (Figure 2f). We searched for EGFR mutations for exons 18, 19, 20, and 21 in the genomic DNA from the adenocarcinoma in situ, but none of them were found (data not shown).

As an adjuvant therapy, the patient has taken orally tegafur-uracil 300 mg/day since 3 months after the resection operation. Until now, she has not shown any signs of relapse or adverse effects. Eleven months after the operation, neither chest CT, nor bone scintigraphy, nor brain magnetic resonance imaging did show any signs of relapse or metastasis.

## Conclusion

In this report, we described a case of synchronous bilateral lung cancers with EML4-ALK positive adenocarcinoma in the right lung and adenocarcinoma in situ in the left, which was EML4-ALK negative. To our knowledge, this is the first report of a case of synchronous lung adenocarcinomas of this combination.

A number of cases of synchronous multiple lung adenocarcinomas were reported previously. The most frequent components in those cases were well differentiated adenocarcinoma with mixed bronchioloalveolar pattern [11]. Graziano et al. [12] reported a case of synchronous bilateral adenocarcinoma in situ of the lung with distinct mutations of EGFR gene. Some of these cases were associated with atypical adenomatous hyperplasia [13,14]. Adenocarcinoma in situ of the lung was frequently associated with EGFR gene mutation [15,16]. There is a case of multiple adenocarcinomas in situ of the lung having the same mutation of EGFR in common [17].

In our case, although the bilateral adenocarcinomas have bronchioloalveolar pattern in common, the right lung cancer was EML4-ALK positive while the left one was not. Furthermore, EGFR gene mutations were not detected in the exons 18 to 21 in “pure” bronchioloalveolar adenocarcinoma in the left lung. Over 90% of EGFR mutations were reported to be localized in these 4 exons [18]. Thus, it is unlikely that the adenocarcinoma in situ in the left lung has EGFR mutation. Interestingly, Togashi et al. recently identified KLC1-ALK fusion gene in a case of adenocarcinoma in situ of the lung [19]. However, in our case, the adenocarcinoma in situ of the left lung was immunohistochemically negative for ALK. Therefore, it is unlikely that the left lung cancer is ALK fusion-positive. The right tumor and the left one had lepidic growth pattern in common, although the former was EML4-ALK positive, while the latter negative. The relationship between genetic alterations and histology is intriguing and it will be interesting to compare gene expression profiling of both tumors.

The fact that the tumor of the right lung and that of the left lung harbor different genetic alterations will be useful for the follow-up of this patient. The adenocarcinoma in situ of the left lung was 15×10 mm of size and pathologically at stage 0 (TisN0M0). Therefore, the possibility of presenting metastasis in the following five years is as low as nearly 0%. On the other hand, the EML4-ALK-positive tumor of the right lung was an invasive cancer. Thus, if any metastasis or relapse occurs in the future in this patient, it is more likely that the relapse derives from EML4-ALK-positive cancer of the right lung. After the confirmation that the metastasis or relapse harbor EML4-ALK translocation, ALK inhibitor such as crizotinib will be the first choice. EML4-ALK fusion gene occurred around 3% of “non-smoker” adenocarcinomas of the lung [7]. In addition to the EML4-ALK fusion, not only other fusion partner for ALK gene, but also novel kinase gene fusions have been discovered in a subset of lung adenocarcinoma [7-9]. It was reported that these kinase gene fusion-positive lung adenocarcinomas have some histological correlates or surrogates including mucinous cribriform pattern [7]. However, as our case report illustrated, kinase gene fusion-positive lung adenocarcinomas may show different histology other than mucinous cribriform pattern. Not only tumor histology, but also patients’ sex, age, smoking habit, and so on, should be considered to suspect the involvement of kinase gene fusion in lung cancers. Although rarer than lung cancer with EGFR mutation, the identification of kinase gene fusions, including EML4-ALK, in lung cancer leads to molecularly-targeted therapy with kinase inhibitors. The identification has important implication for tractable therapy and predictable prognosis.

## Consent

Written informed consent was obtained from the patient for publication of this Case report and any accompanying images. A copy of the written informed consent is available for review by the Editor-in-Chief of this journal.

## Abbreviations

EGFR: Epidermal growth factor receptor; EML4: Echinoderm microtubule-associated protein like 4; ALK: Anaplastic lymphoma kinase; CT: Computerized tomography; HE: Hematoxylin and eosin; FISH: Fluorescent in situ hybridization.

## Competing interests

All the authors state no competing interests.

## Authors’ contribution

IM, Kengo Takeuchi, KU, Kazuhiro Teramura, and SH participated in the pathological final diagnosis of the case, and prepared and edited the manuscript. In particular, Kengo Takeuchi performed the FISH analysis. SM and MK were responsible for the preoperative endoscopic examination and the operations, and helped IM and SH in preparation of the manuscript. All authors read and approved the final manuscript.

## Pre-publication history

The pre-publication history for this paper can be accessed here:

http://www.biomedcentral.com/1471-2466/13/25/prepub
